# Design of a controlled trial to evaluate the effectiveness of Supportive Parenting (‘Stevig Ouderschap’): an intervention to empower parents at increased risk of parenting problems by providing early home visits

**DOI:** 10.1186/s40359-015-0104-1

**Published:** 2015-12-30

**Authors:** E. M. B. Horrevorts, A. van Grieken, S. M. L. Broeren, R. Bannink, M. B. R. Bouwmeester-Landweer, E. Hafkamp-de Groen, Hein Raat

**Affiliations:** Department of Public Health, Erasmus MC University Medical Center Rotterdam, P.O. Box 2040, 3000 CA Rotterdam, The Netherlands; Vereniging Stevig Ouderschap, Oudewater, The Netherlands; Rivas Zorggroep, P.O. Box 90, 4200 AB Gorinchem, The Netherlands

**Keywords:** Study design, Controlled trial, Parenting problems, Supportive Parenting, Prevention, Nursing, Early home visits

## Abstract

**Background:**

In the Netherlands, 15 % of all families with children under the age of 13 years deal with significant parenting problems. Severe parenting problems may lead to adverse physical, cognitive, and psychosocial outcomes for children, both in the short and long run. The intervention Supportive Parenting (in Dutch: “Stevig Ouderschap”) is a preventive program, which aims to reduce the risk of (developing) parenting problems among parents at risk of these problems. The intervention consists of six additional home visits by a Youth Health Care nurse during the first 18 months after childbirth and is focusing on the following elements of parental empowerment: activating social networks, increasing parenting skills and supporting parent(s)/caregiver(s) in getting grip on their own life.

**Methods and design:**

A controlled trial is performed in two regions in the Netherlands. An intervention group receives the intervention Supportive Parenting, and a control group receives ‘care-as-usual’. Parents in both the intervention and control group fill out three questionnaires focusing on various elements of empowerment (social support, parenting skills, self-sufficiency and resilience), behavioral and emotional problems of the child. The effects of the intervention will be evaluated at child age 1–3 months (baseline) and child age 18 months by comparing the outcomes between the intervention group and the control group on the primary outcomes. Additionally, interviews and focus group interviews will be held to identify factors, which hinder or stimulate a wider implementation of the intervention Supportive Parenting.

**Discussion:**

It is hypothesized that parents at increased risk of parenting problems who receive the intervention Supportive Parenting during the first 18 months after childbirth, will have enhanced their social support networks and parenting skills, increased their self-sufficiency and strengthened resilience compared to at risk parents receiving care-as-usual. Additionally children of parents from the intervention group will display less parent-reported behavioral and emotional problems.

**Trial registration:**

Netherlands Trial Register NTR5307. Registered 16 July 2015.

## Background

More than one third of Dutch parents have worried about parenting or the development of their children. More than half of these parents have sought help or advice outside their family or friends for their concerns [1]. These worries are normal and part of parenting [2].

It becomes more problematic when parents experience a discrepancy between how they would wish to raise their child(ren) and their actual parenting situation, and they do not have the means (anymore) to overcome this discrepancy (e.g. they do not know where to seek help or advice). This is what might be referred to as a parenting problem [3].

Kousemaker et al. [4] distinguishes three types of situations in which parenting problems occur, namely a mildly problematic parenting situation (i.e. parenting tasks are not always performed in an effective way and parents do not always have answers to their parenting questions), a moderately problematic parenting situation (i.e. parenting tasks are not performed in an effective way and parents do not have answers to their parenting questions) and a severely problematic parenting situation (parenting style is characterized by ineffectiveness, inconsistency, and excessive actions such as child abuse or neglect).

In the Netherlands, 15 % of all families with children under the age of 13 years deal with problematic parenting situations [1]. Of this 15 %, 10 % deals with a mildly problematic parenting situation, 4 % deals with a moderately problematic parenting situation, and 1 % deals with a severely problematic parenting situation.

A severely problematic parenting situation may lead to adverse physical, cognitive, and psychosocial outcomes for children, both in the short and long run [5–7]. Interventions can contribute to the prevention of these problematic parenting situations.

In the Netherlands, a system for monitoring children’s health and development, and for providing health promotion and disease prevention at set ages from birth onwards is available: i.e. preventive Youth Health Care. It is offered nation-wide and free of charge [8]. Participation is voluntary and the attendance rate during the first months after childbirth is about 95–100 %. During Youth Health Care visits, growth and development of the child are assessed [8, 9]. The Youth Health Care is committed to counsel parents regarding parenting skills and to promote healthy development and growth for all children [9]. Therefore, the Youth Health Care provides an opportunity to contribute to prevention, early detection, and offering interventions to parents with parenting problems.

The intervention Supportive Parenting (in Dutch: “Stevig Ouderschap”) is a theoretically well-founded intervention that aims to reduce the risk of parenting problems among parents at risk of these problems (parents with low social support, psychosocial problems, drug/alcohol use, negative feelings towards pregnancy, problematic history and/or a preterm child or child with low birthweight) [10]. Currently, 51 % of Youth Health Care centers in the Netherlands use the program [11, 12]. Supportive Parenting is based on the theories of Belsky [13–15], Newberger [16] and Baartman [17] and consists of six home visits by a Youth Health Care nurse during the first 18 months after childbirth. During the home visits the focus lies on the empowerment of parents by activating their social networks, increasing parenting skills and supporting parent(s)/caregiver(s) in getting grip on their own life.

Until now, only one study [18] has evaluated the effectiveness of Supportive Parenting on the psychosocial development of the child, parental expectations, social support, alternative punishment methods, and empathy. Bouwmeester-Landweer et al. [18] showed positive, statistically significant, effects on parental expectations and the psychosocial development of children of parents participating in the Supportive Parenting intervention. Effects of the intervention Supportive Parenting on the empowerment of parent(s)/caregiver(s) are unknown.

### Objective

A controlled trial is performed to investigate the effectiveness of the Supportive Parenting intervention in empowering parent(s)/caregiver(s) who are at risk of parenting problems in terms of social support, parenting skills, resilience, and self-sufficiency. Furthermore, we will explore which parent, child, and nurse characteristics are related to the effects of the intervention Supportive Parenting on the empowerment of parent(s)/caregiver(s) at risk of parenting problems. Additionally, interviews and focus group interviews are performed to investigate the factors that promote/hinder a broader implementation (e.g. among parents with older children [>18 months], during pregnancy, among different ethnic groups) of the intervention Supportive Parenting.

### Study hypothesis

The hypotheses of this study are that parents at increased risk of parenting problems who receive the intervention Supportive Parenting during the first 18 months after childbirth, have enhanced their social support network and parenting skills, increased self-sufficiency and strengthened resilience compared to at risk parent(s)/caregiver(s) receiving care-as-usual at child age 18 months. Additionally children from parents of the intervention group will display less parent-reported behavioral and emotional problems at child age 18 months.

## Methods and design

### Study design

A controlled trial is performed with an intervention group and a control group (‘care-as-usual’) in two regions in the Netherlands.

The inclusion of participants started shortly after childbirth. The effects of the intervention on parental empowerment and behavioral and emotional problems of the child will be evaluated at child age 1-3 months (baseline) and child age 18 months by comparing the outcomes between the intervention group and the control group.

Data collection started in January 2014 and will continue until January 2016. This study has received approval by the Medical Ethics Committee of Erasmus MC (MEC-2013-568).

### Procedure

An opportunity sample of two preventive Youth Health Care centers (CJG Rijnmond and Rivas Zorggroep) in two regions of the Netherlands participated in this study.

Nineteen of the 27 care teams of the Youth Health Care center CJG Rijnmond participated as intervention group. These locations offer the intervention Supportive Parenting to parents at risk of parenting problems as part of their regular youth health care.

The care team in the area Goerree-Overflakkee of CJG Rijnmond and all 19 preventive Youth Health Care teams of Rivas Zorggroep participated as control group. At these teams, regular youth health care is offered, the intervention Supportive Parenting is not part of this regular care. Regular care consists of the regular well-child visits at set ages.

### Participants

**Fig. 1:**
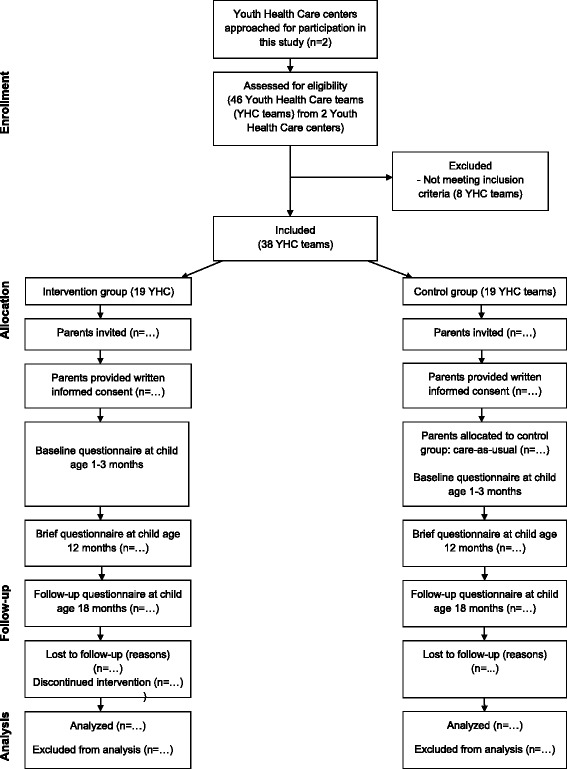
Flow chart of the parents' participation

Between January and September 2014 parents and their children belonging to one of the participating Youth Health Care teams are eligible to participate in the study. Parents in both research groups can only participate in the study if they have at least basic Dutch language skills. The inclusion procedure of the intervention and control condition is described below. The study design and participant flow chart are shown in Fig. 1.

#### Inclusion procedure for the intervention group

As part of the regular well-child visits, a Youth Health Care nurse visits parent(s)/caregiver(s) at home 5–14 days after childbirth. During this visit the Youth Health Care nurse together with the parent(s)/caregiver(s), completes a risk assessment (the Supportive Parenting Questionnaire) to evaluate whether parent(s)/caregiver(s) are at risk of parenting problems. The risk assessment uses a score to identify parents at risk for parenting problems, Youth Health Care nurses compute this score during the visit. At-risk parents are offered the intervention Supportive Parenting.

For this study, the nurse informs these at-risk parent(s)/caregiver(s) about the study and invites parents to participate. The nurse provides the parents with an information leaflet, an informed consent form and the baseline questionnaire of the study. Parent(s)/caregiver(s) are requested to return the completed informed consent form and baseline questionnaire to the researchers in a pre-paid envelope.

#### Inclusion procedure for the control group

In the control group, as part of the regular well-child visits, a Youth Health Care nurse visits parent(s)/caregiver(s) at home 5–14 days after childbirth. The Youth Health Care nurse informs all parent(s)/caregiver(s) about the study and invites them to participate. The nurse provides parents with an information leaflet, an informed consent form and baseline questionnaire of the study. Parent(s)/caregiver(s) are requested to return the completed informed consent form and baseline questionnaire to the researchers in a pre-paid envelope.

After receiving the informed consent form and baseline questionnaire, the researchers compute the parenting problem risk score for all parents using the Supportive Parenting Questionnaire which is included in the baseline questionnaire. Parents at risk for parenting problems participate in the control group. These at risk parents in the control group receive care-as-usual. All other parents are excluded from the control group.

### Intervention supportive parenting

The intervention Supportive Parenting aims to reduce the risk of parenting problems.

Parent(s)/caregiver(s) of newborn children, who are at risk of parenting problems, based on an assessment of risk factors through “the Supportive Parenting Questionnaire”, are offered the intervention. The Supportive Parenting Questionnaire is also based on the theories of Belsky [13–15], Newberger [16] and Baartman [17] and assesses problematic prior history of the parent(s)/caregiver(s) (experience of maltreatment in their own youth or current family; psychological disorders), risk factors of the parent(s)/caregiver(s) (drug and/or alcohol use; negative feelings towards pregnancy; age <19 years of age), risk factors of the child (preterm; low birthweight), risk factors in the social context of the parent(s)/caregiver(s) (single parent; social isolation; low spousal support) and risk factors observed by the Youth Health Care nurse. The main aim of the intervention is to increase parental awareness with regard to the impact of the factors assessed by the Supportive Parenting Questionnaire, on their current daily life and to provide parents with tools to cope with these factors.

The intervention Supportive Parenting consists of six 90 min home visits during the first 18 months after childbirth and focuses on the following elements of parental empowerment: activating social networks, increasing parenting skills and supporting parent(s)/caregiver(s) in getting grip on their own life. A preventive Youth Health Care nurse provides the six home visits of approximately 90 min each. There is one home visit every three months, but according to parents’ needs and preferences there can be more visits during the first months after birth or more visits at the end of the intervention. A home visit consists of a fixed part and a flexible part. During the fixed part the following topics are discussed: handling of/coping with developmental history of the parents, experience of parenthood, expectations with respect to the development of the child, social support and professional support for the family. Additionally during every visit information is given about the different developmental stages of children and the corresponding specific parenting tasks. The flexible part is client-centered. Empowering experiences as well as worrisome experiences are addressed. Parents are asked to come up with ways to improve worrisome aspects of their family-life. The topics of the flexible part are chosen by the parent(s)/caregiver(s) [19]. The intervention is voluntary and parents can indicate to the Youth Health Care nurse if they would like to discontinue the intervention.

The Youth Health Care nurses who provide the home visits have a vast experience in Youth Health Care and have received additional training for the intervention Supportive Parenting. The nurses have the necessary knowledge to provide parent(s)/caregiver(s) with information about health- and development-related issues. However, the nurses are not equipped to provide psychotherapeutic treatment or family therapy and therefore refer to more extensive treatment if deemed necessary [19].

### Control group

Parent(s)/caregiver(s) in the control group of the study receive ‘care-as-usual’ as provided by the Youth Health Care Centers. Parents are invited to visit the Youth Health Care centers for regular well-child check-ups offered by preventive Youth Health Care at set ages (12 check-ups in the first 18 months after childbirth). During these check-ups of twenty minutes, the child’s growth and development are monitored and common advice regarding parenting, development, and growth of children is given (e.g. oral information and generic information leaflets). If needed, parents can be referred to specialized professional care (e.g. social work or medical care).

### Data collection

Data from parent(s)/caregiver(s) in both research groups will be collected at child age 1–3 months (i.e. baseline), at age 12 months (a brief questionnaire) and 18 months (i.e. follow-up). Parents receive self-report questionnaires assessing demographic characteristics, child characteristics (e.g. gender, preterm, low birthweight) and outcomes. Furthermore, nurse characteristics (e.g. personal and work-related) and the working alliance between parent and nurse are assessed by a self-report questionnaire as well. All questionnaires consist of evidence-based instruments, which are described in the measurements section.

Data are handled according to the guidelines of the Dutch Data Protection Authority [20].

### Measurements

#### Primary outcome measurements

The primary outcomes of this study are various elements of empowerment: social support, parenting skills, self-sufficiency, resilience and behavioral and emotional problems of the child at 18 months.

Social support and parenting skills are measured by the Parenting Stress Questionnaire (in Dutch: Opvoedingsbelasting vragenlijst) [21] and the Family Functioning Questionnaire (in Dutch: Vragenlijst Gezinsfunctioneren Ouders) [22]. The Parenting Stress Questionnaire consists of 34 items. Each item is accompanied by a 4-point response scale with 1 = *not true*, 2 = *somewhat true*, 3 = *quite true*, and 4 = *very true.* Five subscales are computed: problems in parent-child relation (six items), problems with parenting (seven items), depressive moods (seven items), role limitations (six items) and health problems of the parent (eight items). Considering the age of the children in this study at baseline, all items of the subscale “problems with parenting” were not included in the baseline questionnaire because the items assess parenting factors that are not applicable to newborn children. Subscale scores are calculated by summing the individual items belonging to a subscale and thereafter converting the subscale scores into T-scores, using the Dutch reference values. For all subscales, scores between T = 30–65 indicate that there are no problems, scores between T = 66–69 indicate moderate problems, and scores of T= > 69 indicate serious problems.

The Family Functioning Questionnaire consists of 28 items. Each item is accompanied by a 4-point response scale with 1 = *not true*, 2 = *somewhat true*, 3 = *quite true*, and 4 = *very true*. Five subscale scores are computed: basic care of the child (seven items), parenting (seven items), social contacts (five items), experience of parent’s own childhood (four items) and partner relation (five items). Considering the age of the children in this study at baseline, no items of the subscale “parenting” were included in the baseline questionnaire because the items assess parenting factors that are not applicable to newborn children. Subscale scores are calculated by the summing the individual items belonging to a subscale and thereafter converting the subscale scores into T-scores, using the Dutch reference values. For all subscales, a score between T = 0–31 indicates problems.

Self-sufficiency is measured by the Empowerment Questionnaire (EMPO) *parents, version* 2.0 (in Dutch: Vragenlijst Empowerment (EMPO) *ouders, versie* 2.0) [23]. The questionnaire consists of 27 items. Each item is scored on a 5-point scale with 1 = *strongly disagree*, 2 = *disagree*, 3 = *neither agree, nor disagree*, 4 = *agree*, and 5 = *strongly agree*. Items can be allocated to three subscales: perceived competence as a person (eight items), perceived competence as a parent (seven items) and utilization of competence (12 items). Considering the age of the children in this study at baseline, five items (three items of the subscale “perceived competence as a parent” and two items of the subscale “competence utilization”) were not included in the baseline questionnaire because the items assessed factors that are not applicable to newborn children. Subscale scores are calculated by the sumscores of the individual items belonging to that subscale and thereafter converted into scores between 1 and 10. A low score indicates problems.

Resilience is measured by the Resilience Scale – Dutch version [24]. The questionnaire consists of 25 items. Each item is scored on a 4-point scale with 1 = *strongly disagree*, 2 = *disagree*, 3 = *agree*, and 4 = *strongly agree*. Items can be allocated into two subscales: personal competence (17 items) and acceptance of self and life (eight items). The minimum total score is 25, the maximum total score is 100 with higher scores indicating higher resilience.

Behavioral and emotional problems of the child at 18 months is assessed by the Child Behavior Checklist (CBCL) for ages 1½–5 [25]. The CBCL consists of 99 items. Each items is scored on a 3-point scale with 0 = *not true*, 1 = *somewhat or sometimes true*, and 2 = *very true or often true*. The scoring gives a summary profile (internalizing, externalizing, and total problem scores), a syndrome profile (emotionally reactive, anxious/depressed, somatic complaints, withdrawn, sleep problems, attention problems, and aggressive behavior) and five scales (affective problems, anxiety problems, pervasive developmental problems, attention deficit/hyperactive problems, and oppositional defiant problems) oriented at the Diagnostic and Statistical Manual for Mental Disorders (DSM). A T-score of ≥63 for summary scales and ≥70 for syndrome and DSM-oriented scales, are considered clinically significant. Scores between 60 and 63 for summary scales or between 65 and 70 for syndrome and DSM-oriented scales are considered as borderline clinically significant. Scores under 60 or 65 are considered non-clinical [25].

#### Other measures

Parent characteristics that are assessed include various demographic factors (age, country of birth, income in euros, educational level, employment situation, and family structure). Additionally, the intervention group completes questions on the amount of home visits they have received, and their satisfaction with the intervention, to check for adherence to intervention protocols. Each questionnaire contains an open space for parents to write down comments and questions with regard to the study and the intervention.

Professional characteristics that are assessed are personality, measured by the Brief HEXACO Inventory [26] and work-related factors, measured by the Utrechtse Burnout Scale [27]. Both the Youth Health Care nurse and parents complete the Working Alliance Inventory (in Dutch: Werkalliantie Vragenlijst [WAV]) to assess the quality of the relation between parent and nurse [28].

Child characteristics which are assessed are gender, preterm, low birthweight and temperament. Temperament of the child is measured by an adapted version of six scales of the Infant Behavior Questionnaire – Revised (IBQ-R) [29] as used in a study by Roza et al. [29]. The IBQ-R asks parents to rate the frequency of specific behaviors observed during the past week. The adapted version of the IBQ-R uses six of the 14 scales because these scales are judged by Roza et al. [30] to be the most important for the later prediction of the most prevalent behavioral problems in children (e.g. anxiety, aggressive behavior and attention problems). The six scales in the adapted version include Activity Level, Distress to Limitations, Fear, Duration of Orienting, Recovery from Distress and Sadness. Based on the results of the pilot study carried out by Roza et al. [30], the original 7-point scale was adapted to a 3-point scale with 0 = *never present*, 1 = *sometimes present* and 2 = *often present.* This was done, because respondents rarely used the extreme points of scales. Higher scores on the scales, except on the Falling Reactivity scale, indicate more difficult behavior. The scores for each scale were calculated by dividing the sum of the items by the number of completed items [30].

### Power of the study

Two Youth Health Care centers participate in the study. Their teams invited 313 parents (for the intervention group) and 2346 parents (for the control group). Taking into account informed consent by 50 % and eligibility of 10 % to participate in the study for the control group, we expect data of 157 parents in the intervention group and 117 parents in the control group.

With the use of continuous measures and assuming a standard deviation of 1.00 in both groups, a power of 0.80 and an alpha of 0.05, these group sizes are sufficient to demonstrate a significant difference of 0.35 between the intervention group and the control group. This is appropriate to indicate relevant effects [31, 32].

### Statistical analysis

Descriptive statistics will be used to describe the characteristics of the sample. Linear regression will be used for the evaluation of continuous outcomes and logistic regression for dichotomized outcomes. Research condition (i.e. intervention or control group), will be entered in the model as the independent variable. Where relevant, models will be corrected for the baseline measurements (data of baseline questionnaire) and for potential confounders (age of child and parent, educational level of parents and ethnic background). Additionally, moderation of intervention effects by sociodemographic characteristics (educational level, income and ethnic background) is explored by adding an interaction term to the regression model.

Missing data on the questionnaires will be handled according to the questionnaire protocol.

### Interviews and focus group interviews

Additionally, interviews [one-on-one] and focus group interviews [with multiple respondents] with Youth Health Care nurses and parents are performed to investigate the factors that promote and/or hinder a wider implementation of the intervention Supportive Parenting. The interviews and focus group interviews will be semi-structured [33] and focus on which aspects of the intervention Supportive Parenting parents and Youth Health Care nurses appreciate, which aspects should be further improved, and the perceived effect of the intervention Supportive Parenting on parents’ empowerment. Furthermore, Youth Health Care nurses will discuss opportunities and obstacles for wider implementation of the intervention Supportive Parenting (e.g. among parents of different subgroups, older children).

#### Participants

##### Interviews and focus group interviews with parents

Parent(s)/caregiver(s) participating in the intervention group of the controlled trial, who are finishing or have already finished the intervention Supportive Parenting are invited by email to participate in an interview or focus group interview. In addition, Youth Health Care nurses who provide the intervention invited parents who are not part of the intervention group but are finishing or have already finished the intervention Supportive Parenting to participate in the interviews.

##### Interviews with Youth Health Care nurses

Youth Health Care nurses who provide the intervention are also invited by email to participate in an interview or focus group interview.

## Discussion

Parenting problems may lead to adverse physical, cognitive and psychosocial outcomes in children, both in the short and long run. Interventions such as the intervention Supportive Parenting, can contribute to the prevention of parenting problems. In this controlled trial the effectiveness of the intervention Supportive Parenting in empowering parent(s)/caregiver(s) at increased risk of parenting problems in terms of social support, parenting skills, resilience, and self-sufficiency, is evaluated.

It is hypothesized that parent(s)/caregiver(s) at increased risk of parenting problems, who receive the intervention Supportive Parenting during the first 18 months after childbirth, have enhanced their social support network and parenting skills, increased their self-sufficiency and strengthened resilience compared to at-risk parents receiving care-as-usual. Also parent characteristics (demographic factors) and nurse characteristics (work-related and personal factors) and the working alliance between parent and nurse will be evaluated. Additionally, interviews and focus group interviews are performed. This will provide insights relevant for a wider implementation of Supportive Parenting. Results of the study will be presented and discussed with relevant professionals.

Strengths of the study are that the intervention Supportive Parenting is based on successful international interventions. Effective elements of international parenting interventions such as home visitation and frequency and duration of the home visits are incorporated in the intervention Supportive Parenting. Also, the previous positive effects of Supportive Parenting on the parental expectations and psychosocial development of children of parents participating in the Supportive Parenting intervention, found by Bouwmeester-Landweer et al. [18], are a strength of this study. Furthermore, this study is conducted within the daily practice of the Youth Health Care. The nurses who provide the intervention Supportive Parenting already have experience with this intervention. This allows us to assume that the intervention is performed correctly.

A challenge of this study may be the relative high risk intervention group. Parents at risk of parenting problems are a challenging group to reach and are often hesitant to participate in research [34]. However, through close collaboration with the Youth Health Care centers and the Youth Health Care nurses who provide the intervention Supportive Parenting, it is possible to realize participation of this important group of parents.

In conclusion, this paper describes the design of a controlled trial on the prevention of parenting problems by targeting the empowerment of parent(s)/caregiver(s).

## Abbreviations

EMPO: Empowerment Questionnaire (in Dutch: Vragenlijst Empowerment)
CBCL: Child Behavior Checklist
DSM: Diagnostic and Statistical Manual for Mental Disorders
WAV: Working Alliance Inventory (in Dutch: Werkalliantie Vragenlijst)
IBQ-R: Infant Behavior Questionnaire – Revised
